# Genome Survey Sequencing and Genetic Background Characterization of *Ilex chinensis* Sims (Aquifoliaceae) Based on Next-Generation Sequencing

**DOI:** 10.3390/plants11233322

**Published:** 2022-12-01

**Authors:** Peng Zhou, Jiao Li, Jing Huang, Fei Li, Qiang Zhang, Min Zhang

**Affiliations:** 1 Jiangsu Academy of Forestry, 109 Danyang Road, Dongshanqiao, Nanjing 211153, China; 2 Co-Innovation Center for Sustainable Forestry in Southern China, Key Laboratory of State Forestry and Grassland Administration on Subtropical Forest Biodiversity Conservation, College of Biology and the Environment, Nanjing Forestry University, Nanjing 210037, China

**Keywords:** genome survey, next-generation sequencing, flow cytometry, microsatellite, *Ilex chinensis* Sims

## Abstract

*Ilex chinensis* Sims. is an evergreen arbor species with high ornamental and medicinal value that is widely distributed in China. However, there is a lack of molecular and genomic data for this plant, which severely restricts the development of its relevant research. To obtain the whole reference genome, we first conducted a genome survey of *I. chinensis* by next-generation sequencing (NGS) to perform *de novo* whole-genome sequencing. As a result, our estimates using k-mer and flow cytometric analysis suggested the genome size of *I. chinensis* to be around 618–655 Mb, with the GC content, heterozygous rate, and repeat sequence rate of 37.52%, 1.1%, and 38%, respectively. A total of 334,649 microsatellite motifs were detected from the *I. chinensis* genome data, which will provide basic molecular markers for germplasm characterization, genetic diversity, and QTL mapping studies for *I. chinensis*. In summary, the *I. chinensis* genome is complex with high heterozygosity and few repeated sequences. Overall, this is the first report on the genome features of *I. chinensis*, and the information may lay a strong groundwork for future whole-genome sequencing and molecular breeding studies of this species.

## 1. Introduction

*Ilex chinensis* Sims. is an evergreen arbor species belonging to the genus *Ilex* in the family Aquifoliaceae. It is widely distributed in the south of Qinling Mountains and Huai River of China, as one of the native dominant species in evergreen broadleaved forests [1,2]. Compared with other hollies, *I. chinensis* not only has strong ornamental value, but also is a commonly used traditional Chinese medicine herb, the dried leaves of which are known in China as ‘Si ji qing’, used to treat acute laryngopharyngitis, bronchitis, dysentery, burns, and as an external treatment for skin ulcer in China [3]. For its excellent performance and great breeding value, it has been identified as a precious tree in China.

The current research on *I. chinensis* mainly focuses on the chemical composition and pharmacological effects, while the genetic information of *I. chinensis* has remained largely unknown [4,5,6], although some studies have performed transcriptome analysis [7], genetic diversity analysis based on SSR markers [2], and complete chloroplast genome sequencing [8]. Thus far, most genomic research on the *Ilex* genus is focused on very few commercially important species, including *I. latifolia* [9], *I. asprella* [10], and *I. polyneura* [11], whereas few studies have been reported for *I. chinensis*. The lack of a reference genome for *I. chinensis* leads to limited enhancement in molecular biology and breeding programs.

The genome contains all the information controlling biological characters and ultimately determining the transmission of genetic material [12,13,14]. Genome sequencing has been an important step to decipher the genetic structure and accelerate genetic improvements in traits of interest in eukaryotes [15,16]. Exploring the genes related to the effective components and excellent agronomic characters of plants, and analyzing the metabolic pathways and regulatory mechanisms from the genome-wide level, can lay the foundation for the improvement of medicinal plant varieties and the protection of genetic resources [14,17]. Owing to recent advances in DNA sequencing techniques, draft genomes have been assembled for many plant species as resources for genomic and genetic research efforts [18,19,20]. However, the genomes of some plant species, particularly tree species and medicinal plants, are highly heterozygous, and have a complex genetic background [12,21,22]. Therefore, it is imperative to obtain basic knowledge on the genome structure of the target material before large-scale sequencing of this species [12,18].

Genome surveys, which use next-generation sequencing (NGS), have been proven to be important strategies for exploring genomic information for plants, especially for non-model plants, by yielding a large amount of genomic data in a rapid, cost-effective manner [15,23]. Genomic data from genome surveys not only can provide useful information on genome structure, such as an estimation of genome size, heterozygosity levels, and repeat contents, but also establish a genomic sequence resource from which molecular markers can be developed [24,25,26]. In this study, the NGS method was used to conduct *de novo* whole-genome sequencing of *I. chinensis*. In combination with flow cytometry, the genome size and characteristics of *I. chinensis* were obtained, which may provide a basis for future whole-genome sequencing, and will be conducive to key genetic analysis for effective component synthesis as well as the breeding of this species.

## 2. Results

### 2.1. Genome Size Estimation by Flow Cytometry

**Figure 1:**
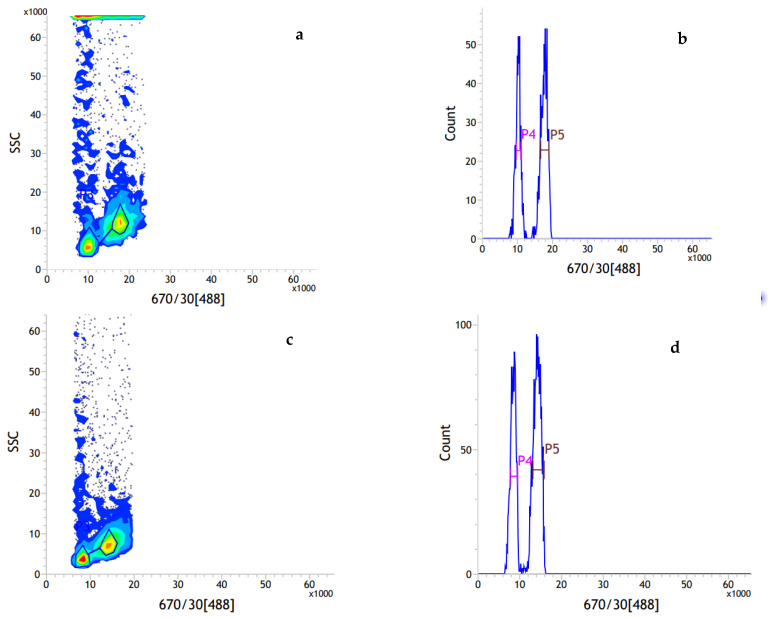
The result of FCM analysis for *I. chinensis* samples simultaneously processed with rice: (**a**,**b**) the representative female sample; (**c**,**d**) the representative male sample. Scatterplot with side scatter (SSC) versus PI fluorescence with manually drawn polygon gate (**a**,**c**), histogram of relative fluorescence intensity derived from nuclei isolated from rice and *I. chinensis* processed simultaneously (**b**,**d**). Peak 4 represents G_0_/G_1_ nuclei of rice, peak 5 represents G_0_/G_1_ nuclei of *I. chinensis* sample.

The flow cytometric analysis yielded a high-resolution histogram (Figure 1). The mean CVs were 4.05% and 4.42% for *I. chinensis* and the internal standard rice, respectively (Table 1). The average genome size for *I. chinensis* male and female samples was estimated to be approximately 660 ± 10 Mb. The genome size of male samples was estimated to be larger than that of the female samples, with 665 ± 12 and 656 ± 6 Mb, respectively. A t-test suggested that there was no significant difference between male and female samples in genome size (*p* > 0.05), which indicated that the differences in genome size between sex in *I. chinensis* might be small enough to be undetectable using flow cytometry, or might be due to the accuracy of the current method.

### 2.2. Sequencing and Quality Evaluation of Ilex chinensis

**Figure 2:**
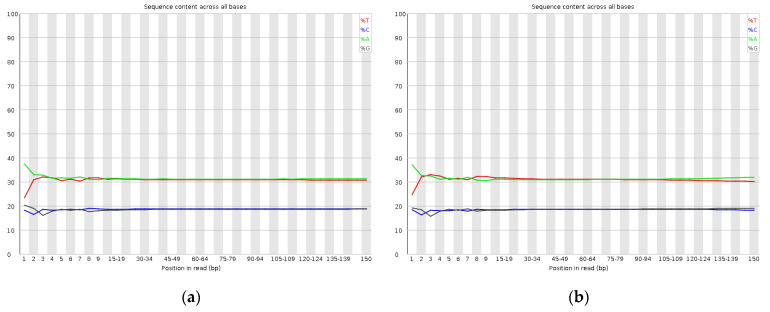
Distribution figure of AT and GC content: (**a**) read 1 AT and GC content distribution; (**b**) read 2 AT and GC content distribution. Different colors represent different base types.

The libraries of paired-end sequencing with 350 bp short inserts of *I. chinensis* were constructed. A total of 44.813 Gb of raw data, which was an approximately 67.90-fold coverage of the estimated genome size, were generated by the DNBSEQ-T7 sequencing platform. After filtering and correction, a total of 44.645 Gb of *I. chinensis* clean bases were obtained with the Q20 and Q30 values of 95.275% and 87.84%, respectively (Table 2), which indicated that the high-throughput sequencing of *I. chinensis* was highly accurate. In total, 91.31% of the reads were used for assembly. Figure 2 showed the proportion of single bases, which was used to detect whether AT and GC separation was present. It could be seen that the content of A and G and C and T were close. The results demonstrated that the sequencing quality was good.

### 2.3. Genome Size Estimation by K-Mer Analysis

**Figure 3:**
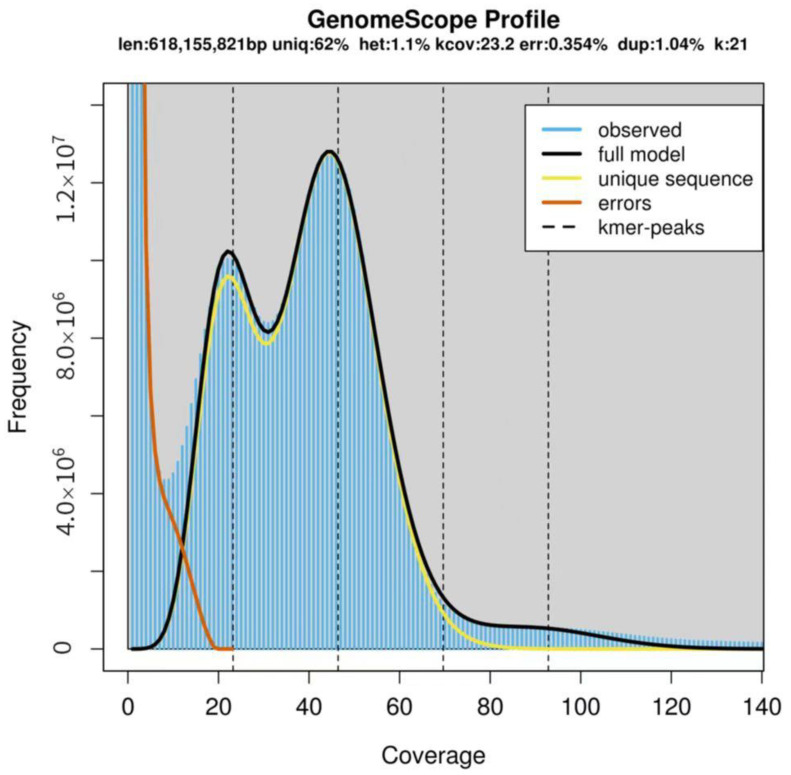
K-mer (k = 21) distribution as calculated by GenomeScope. The *x*-axis is depth and the *y*-axis is the proportion that represents the frequency at that depth divided by the total frequency of all depths. Blue bars represent the observed k-mer distribution; the black line represents the modeled distribution without the k-mer errors (red line) and up to a maximum k-mer coverage specified in the model (yellow line). Abbreviations: len, estimated genome length; uniq, unique portion of the genome (nonrepetitive elements); het, genome heterozygosity; err, the sequencing error rate.

All the clean data were used for k-mer analysis using a K-value of 21, and the obtained results were presented in a frequency distribution graph (Figure 3). The main peak of depth was at 46×, and the estimated genome size of *I. chinensis* was 618 Mb. In addition, the percentage of the number of k-mers after 1.8-fold of the main peak in the total number of k-mers were used to estimate the repeat rate. The heterozygosity rate appeared at a position of half the height of the main peak (23×). The genome heterozygosity rate and sequence repeat rate were calculated to be approximately 1.1% and 38%, respectively. Therefore, the genome of this species belongs to a complex genome with high heterozygosity.

### 2.4. Genome Assembly and Guanine plus Cytosine (GC) Content Analysis

**Figure 4:**
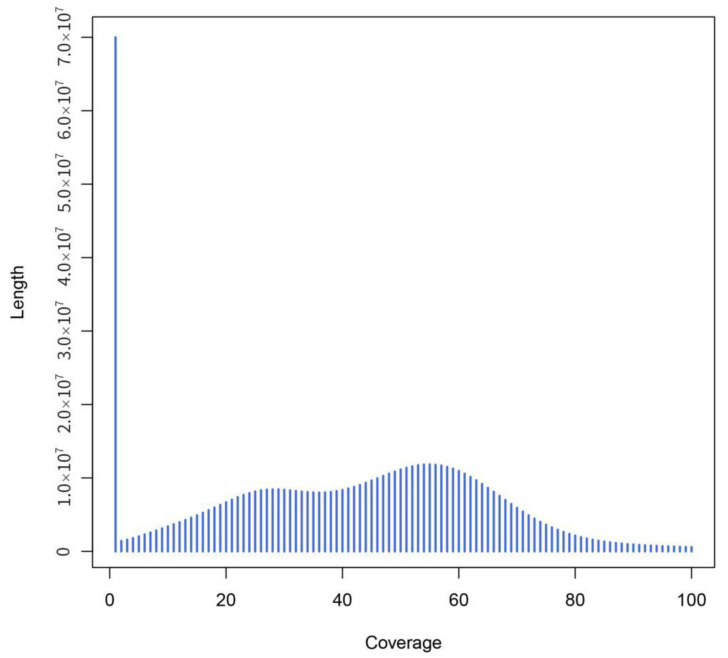
Distribution of scaffold coverage depth and length. The *x*-axis represents the coverage and the *y*-axis is the sequence length.

**Figure 5:**
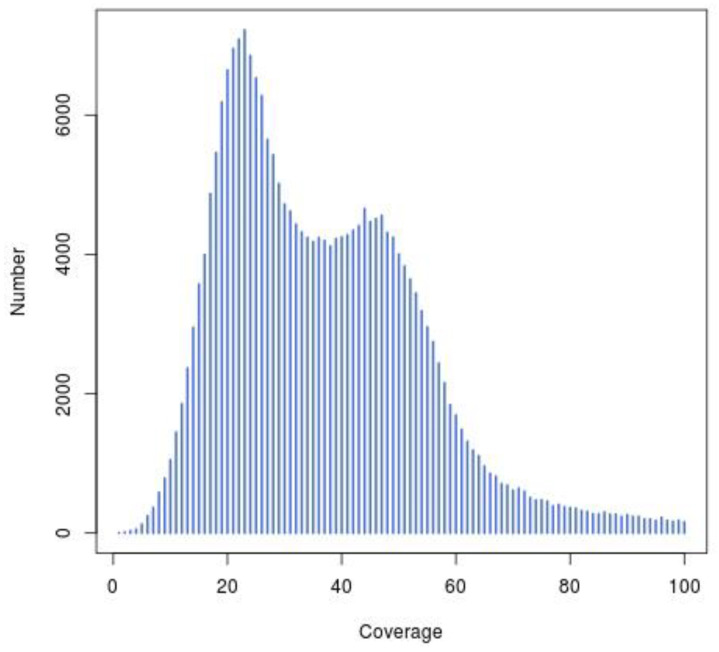
Distribution of scaffold coverage depth and number. The *x*-axis represents the coverage and the *y*-axis is the sequence number.

The total number of raw contigs was 9,683,026, with an N50 of 173 bp. Scaffolds larger than 500 bp were selected to avoid low-quality sequences; consequently, the genome assembly consisted of 250,019 scaffolds, with a total length of 685,140,399 bp, and the scaffolds’ N50 length was 5738 bp (Table 3). The results from Figure 4 and Figure 5 showed significant peaks. It could be determined that the peak value at approximately 50× is a homozygous peak. The peak that was located around half of the x-coordinates in front of the homozygous peak was the heterozygous peak. Therefore, the genome of *I. chinensis* is highly heterozygous and complex.

**Figure 6:**
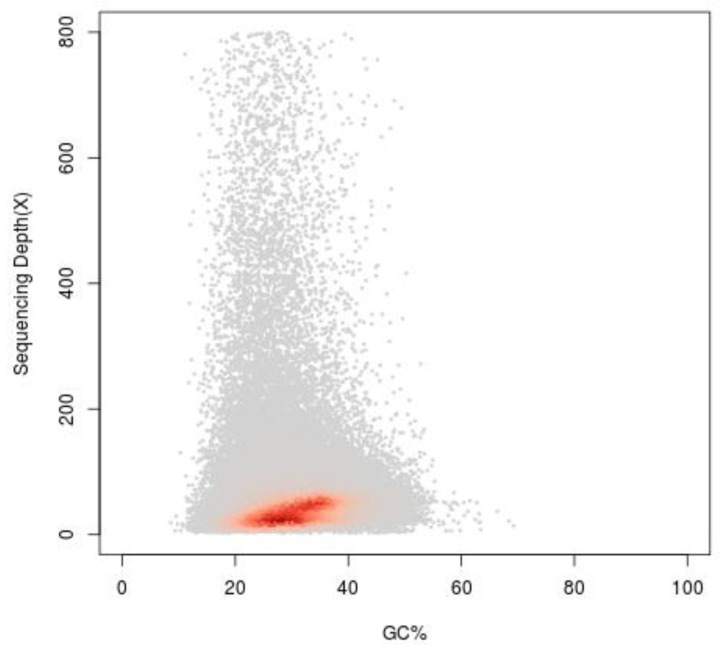
Guanine plus cytosine (GC) content and depth correlation analysis. The *x*-axis represents the GC content and the *y*-axis is the sequence depth.

GC content is one of the causes of the bias in sequencing results. According to the GC depth distribution map (Figure 6), it can be judged whether there is obvious GC bias in the sequencing results, and also determined whether there is bacterial contamination. Then, scaffolds larger than 500 bp were used to build a scatterplot. In Figure 6, the scatterplot showed that the confidence area (shown in red) was around 20–40%, where the average GC content of the *I. chinensis* was 37.52% after calculation. In addition, the GC depth map was divided into two layers (Figure 6), which was mainly due to the high heterozygosity [27].

### 2.5. Identification and Characteristics of Microsatellite Motifs

**Figure 7:**
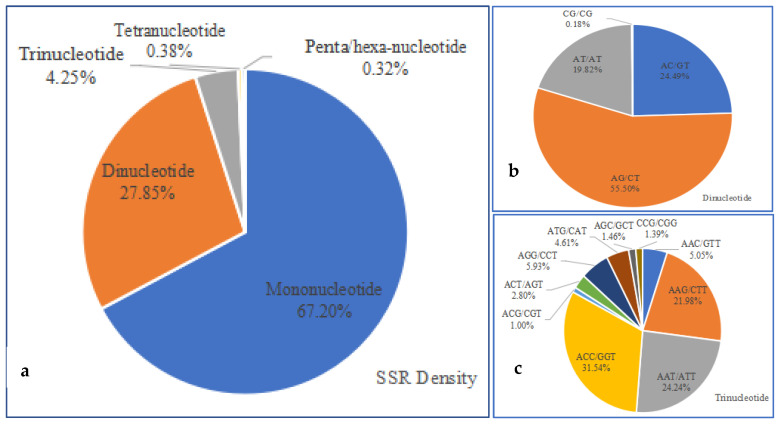
Characteristics of microsatellite motifs: (**a**) frequency of different microsatellite motifs; (**b**) frequency of different dinucleotide motifs; (**c**) frequency of different trinucleotide motifs.

A total of 334,649 microsatellite motifs were detected. Among these microsatellite motifs, the mononucleotide motifs were the most abundant, accounting for 67.20% (224,888) of the total microsatellite motifs, followed by di- (93,211, 27.85%), tri- (14,215, 4.25%), tetra- (1261, 0.38%), penta- (435, 0.13%), and hexanucleotide (639, 0.19%) motifs (Figure 7a). There was a large proportion of both mononucleotide and dinucleotide motifs, which amounted to 95.05%, while the rest amounted to less than 5%. In the dinucleotide motifs, AG/CT accounted for 55.50%, the AC/GT accounted for 24.49%, AT/AT accounted for 19.82%, and CG/CG only accounted for 0.18% (Figure 7b). As for the predominant trinucleotide motifs, the ACC/GGT, AAT/ATT, and the AAG/CTT accounted for 31.54%, 24.24%, and 21.98%, respectively (Figure 7c).

## 3. Discussion

### 3.1. Genome Size of Ilex chinensis

Genome size is one of the important attributes of the genome of an organism, and before commencing genome sequencing, accurate estimations of nuclear genome sizes are prerequisites [28,29,30]. There are several methods to predict the genome size, such as feulgen spectrophotometry, pulsed-field gel electrophoresis (PFGE), and flow cytometry [19]. Though flow cytometry has become a well-recognized technique for predicting genome size before plant genome sequencing due to its low material requirements, short analysis time, and high accuracy, there is still some technical error that will hinder exact quantification [21,31]. With the rapid development of NGS technology, k-mer analysis, which is based on whole-genome sequencing reads, has become an effective and economical method to obtain genome sizes of non-model or emerging species [32], which has been proven successful for many plant species, such as *Metroxylon sagu* Rottboll [19], *Rhododendron micranthum* [14], *Pistacia vera* [15], and *Akebia trifoliata* [33]. Therefore, using both methods to measure genome size can improve the reliability of the results. In this study, the genome size of *I. chinensis* determined by flow cytometry was approximately 660 Mb, and our k-mer analysis using genome survey data suggested a genome size of around 618 Mb, which was close to the results derived from flow cytometry. Together, these data provide robust evidence that the genome size of *I. chinensis* is 618–660 Mb, a value that was slightly smaller than those of other members of the *Ilex* genus family reported previously [9,10,11]. *I. chinensis*, with its small genome size, can often be given priority in performing whole-genome sequencing projects for economic reasons [34].

### 3.2. The Genome Characteristics of Ilex chinensis

GC content was one of three factors found to contribute to sequencing bias on the Illumina sequencing platform. GC content exceeding the normal range (>65% or <25%) will result in reduced coverage in sequencing regions, which can seriously affect genome assembly [35]. In this study, the GC content of *I. chinensis* was medium, which fell within the general range of 30 to 47% for most plants [19,36], and would not influence the quality of the genome sequence during the sequencing process [18,27].

Heterozygosity and repeat rates of genome are critical factors used to determine the quality of genome assembly, and identifying the heterozygosity and repeatability of the sequencing data is conducive to design appropriate assembly strategy [21,25]. The genome of *I. chinensis* was considered a simple repetitive genome [37], which may be due to the small genome size of this species [38]. Repeated sequences are one of the major factors that control the recombination and regulation of structural genes [36], and further investigation is required to understand the functions of repeated sequences in *I. chinensis* [14]. The heterozygosity of sequence data is helpful to find a suitable genomic splicing method, which can be divided into high heterozygosity (≥0.8%) and low heterozygosity (0.5%–0.8%) [37]. Possibly due to the dioecious mating system in the *Ilex* genus [39], the heterozygosity of *I. chinensis,* similar to that of *I. polyneura* (1.18%) within the same family (Aquifoliaceae) [11], was high, and this indicated the high complexity in the genome. The characteristics of the *I. chinensis* genome might impact the accuracy of genome size estimation and the quality of genome assembly. Previous studies have suggested that if the genomic heterozygosity exceeds 1%, the genome is considered to be rather difficult to assemble [12,33]. Therefore, the genome of *I. chinensis* was relatively difficult to assemble by traditional approaches, and the N50 lengths of scaffolds were relatively short (Table 3). However, with the invention of the third-generation sequencing, highly heterozygous genomes have been successfully assembled [40,41].

Considering the complex characteristics of the *I. chinensis* genome and its high heterozygosity, we should adopt the strategy of combining the second-generation and third-generation sequencing, and use the Hi-C technique and BioNano Genomics for supplement to sequence and assemble in the following whole-genome sequencing studies [12,18,42].

### 3.3. Characteristics of Microsatellites in Ilex chinensis

The identification and characterization of repetitive DNA is a fundamental step towards the genetic and biological characterization of plants [25]. A large number of genome-wide SSR loci have provided a foundation for the further construction of high-density genetic maps and the study of the regulatory mechanisms of medicinal ingredients derived from *I. chinensis* [14,43,44]. For most plants, the di- and trinucleotide repeat motif types mostly contribute to the major proportion of SSRs, and a notably small proportion was contributed by tetra-, penta-, and hexanucleotide repeat motif types [15,33,45,46]. In this study, the mononucleotide repeat was the most abundant type, which was similar to many species, such as *Sillago sihama* [47], *Betula platyphylla* [46], and *R. micranthum* [14].

Additionally, the results showed that AG/CT (55.50%) and ACC/GGT (31.54%) were frequent among the di- and trinucleotide repeat SSRs in *I. chinensis*, which differed from those in such species as *Erianthus arundinaceus* (AG/CT, 54.16% and AAT/ATT, 20.23%) [48], *M. sagu* Rottboll (AT/TA, 29.07% and CCG/CGG, 18.1%) [19], *R. micranthum* (GA/TC, 38.63% and CAC/GTG, 12.03%) [14], and *Acer truncatum* Bunge (AT/AT, 71.31% and AAT/ATT, 54.72%) [49]. These differences in distribution frequency of SSR motif types suggest that the duplicated regions in each species may be subject to different selection pressures. The reason for the high polymorphism at these loci needs further investigation.

## 4. Materials and Methods

### 4.1. Plant Materials

**Figure 8:**
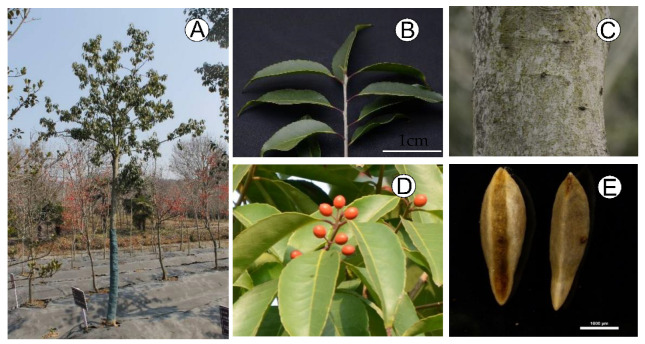
The morphological characteristics of *I. chinensis*: (**A**) the plant; (**B**) the leaves; (**C**) the tree bark; (**D**) the branch with fruits; (**E**) the pyrene.

*Ilex chinensis* (approximately 20 years old) were collected from the national holly germplasm bank of China and planted in the testing grounds of Jiangsu Academy of Forestry (Nanjing, Jiangsu, China). For each sex of *I. chinensis*, young leaf tissue from five individuals was selected in the field for the flow cytometry analysis. Fresh leaves from a single female individual of *I. chinensis* tree (Figure 8) were selected for genome survey sequencing. Total genomic DNA was isolated using a DNA extraction kit (CWBIO, Shanghai, China). DNA integrity was monitored on 1% agarose gel electrophoresis, and the purity was detected via a NanoDrop 2000 (Thermo Fisher, Wilmington, DE, USA). DNA concentration was measured using a Qubit fluorometer (Thermo Fisher, Waltham, MA, USA).

### 4.2. Genome Size Estimation by Flow Cytometry

For each sex of *I. chinensis*, young leaf tissue was collected for flow cytometry analysis. Samples were prepared by slightly modified procedures as described by Kandaiah et al. [50]. Approximately 50 mg of fresh *I. chinensis* leaf tissue with a similar amount of internal standard *Oryza sativa* subsp. japonica cv. Nipponbare (1C = 389 Mb, GC = 43.6 %) [51] were co-chopped in 1 mL pre-cooled Tris dissociation solution with a new razor blade. The mixture was filtered through a 40 µm nylon mesh. The filtrate was centrifuged at 1000 rpm/min for 5 min at 4 °C, and the pellet was stained with 500 μL propidium iodide (PI, 50 µg/mL) stain solution (containing 50 µg/mL RNase A). The resuspension was incubated in the dark at 4 °C for 5 min, and then filtered and loaded onto the BD Influx^TM^ cell sorter (BD, Piscataway, NJ, USA) for detection. The flow cytometer was equipped with an argon ion laser operating at 488 nm. The PI fluorescence was collected by 670 nm FL2 filter. In each sample, at least 5000 nuclei were evaluated. FACS software 1.0.0.650 was used for capturing fluorescent signals and data analysis with the coefficient variation values (CV) of both peaks controlled in 5% [52]. The holoploid genome size of each sample was calculated according to the following formula: Sample genome size = [(sample G_0_/G_1_ peak mean)/(standard G_0_/G_1_ peak mean)] × standard genome size [52]. Variance analysis was carried out using Excel 2013 and SPSS 13.0 with convective detection parameters. A t-test was performed to determine whether differences were present in genome size between male and female samples, and *p* < 0.05 was considered statistically significant.

### 4.3. Genome Sequencing

The genomic paired-end libraries with 350 bp insertions were constructed on an DNBSEQ-T7 sequencing platform using *I. chinensis* following the guidance of the standard procedure (MGI Tech Co., Ltd., Shenzhen, China) at the Compass Agritechnology Co., Ltd. (Beijing, China). In order to ensure the quality of the analysis, we filtered the paired reads that would interfere with subsequent information, the paired reads with adapter contamination, the paired reads with uncertain nucleotides (N) that constitute more than 10% of either read, and the paired reads when low quality nucleotides (base quality less than 5) constitute more than 50% of either read. Clean reads were obtained after filtering and correction of the sequence data.

### 4.4. Estimation of Genome Size, Heterozygosity, and Repeat Rate

K-mer analysis was conducted using Jellyfish [53], and the k-mer histogram generated for K-values of 21 was submitted to the GenomeScope to estimate the genome size, heterozygosity, and repeat rate of the clean reads before genome assembly [54,55]. GenomeScope parameters were as follows: read length 150 bp and max k-mer coverage 1000.

### 4.5. Genome Assembly and Guanine plus Cytosine (GC) Content Analysis

The software SOAPdenovo2 was used for the assembly of the refined MGI PE reads, and k-mer size = 21 was chosen for assembly with default parameters in SOAPdenovo2 to construct the de Bruijn graph. To simplify the de Bruijn graph, the sequences at every bifurcation locus were truncated to obtain the initial contigs. The reads obtained from sequencing all the libraries were aligned to the initially obtained contigs. The connectivity relationship between the reads and the information of the inserted fragment size were used to further assemble the contig into a scaffold and obtain the primary genomic sequence containing Ns. Following this, the filtered reads were aligned to this assembled sequence using SOAP to obtain the base depth. The resultant scaffolds of more than 500 bases in length were chosen. A window size of 10 kb was used for non-repetitive advancement in the sequence and calculation of the mean depth and GC content of every window to generate a GC depth plot.

### 4.6. Identification and Verification of Microsatellite Motifs

Microsatellite identification software (MISA) was used to identify all microsatellite repeat units, conduct an analysis of SSR molecular markers in the DNA sequence, and calculate the location, length, quantity, start sites, and end site of the SSR molecular markers in the scaffold. The minimum numbers of SSRs for mono-, di-, tri-, tetra-, penta-, and hexanucleotides adopted for identification were 10, 6, 5, 5, 5, and 5, respectively.

## 5. Conclusions

In this study, we obtained first insight into the genome features of *I. chinensis*, and this information will help to design whole-genome sequencing strategies. The genomic characteristics of *I. chinensis* include a genome size of about 618–660 Mb, a heterozygosis rate of 1.1%, a repeat rate of 38%, and a GC content of 37.52%. A total of 334,649 microsatellite motifs were detected from the *I. chinensis* genome data. These results and the study dataset may provide a solid foundation for whole-genome sequencing, molecular-marker-assisted breeding, as well as functional genome research on *I. chinensis* in the future.

## Figures and Tables

**Table 1:** Statistics of all samples using flow cytometry.

	Genome Size (Mb) Mean ± SD	CV (%) of Samples	CV (%) of Standard
Female	656 ± 6	4.00	4.19
Male	665 ± 12	4.09	4.64
Average	660 ± 10	4.05	4.42

Abbreviations: CV, the coefficient variation value of peak.

**Table 2:** Sequencing data statistics and quality assessment of *I. chinensis*.

Number of Raw Reads	Raw Base (Gbp)	Number of Clean Reads	Error Rate (%)	Q20 (%)	Q30 (%)	GC Content (%)	Average Quality
298,755,430	44.813	297,637,262	0.354	95.275	87.84	37.52	34.75

Abbreviations: Q20, percentage of bases with quality value ≥ 20; Q30, percentage of bases with quality value ≥ 30.

**Table 3:** Statistics of assembled genome sequences of *I. chinensis*.

	Total Length (bp)	Total Number	Max Length (bp)	Min Length (bp)	N20 Length (bp)	N50 Length (bp)	N80 Length (bp)
Scaffold	685,140,399	250,019	112,652	500	15,137	5738	1663

## Data Availability

The data presented in this study are available on request from the corresponding author.
